# Workaholism, work engagement, and affective commitment: relationships to self-concept levels and work outcomes

**DOI:** 10.3389/fpsyg.2024.1434482

**Published:** 2025-01-03

**Authors:** Marie-Colombe Afota, Véronique Robert, Christian Vandenberghe

**Affiliations:** ^1^Department of Industrial Relations, University of Montreal, Montréal, QC, Canada; ^2^TSM Research, Toulouse School of Management, Université Toulouse Capitole, CNRS, Toulouse, France; ^3^Departement of Management, HEC Montréal, Montréal, QC, Canada

**Keywords:** Workaholism, work engagement, affective organizational commitment, selfconcept, work hours, role overload, depression, emotional exhaustion

## Abstract

As a result of the significant changes in businesses around the globe such as the generalization of remote working and digital transformation, the boundaries between work and private life tend to vanish, causing concerns about whether individuals’ investment in their work could have detrimental effects on their life and health. In such context, the notion of heavy work investment, an umbrella construct that subsumes different forms of investment of the self into the work domain, warrants scholarly attention as it may have both a bright and dark side for individuals. The present study focuses on three forms of heavy work investment, i.e., workaholism, work engagement, and affective organizational commitment, and was aimed at examining their association with three levels of the self-concept, i.e., individual, relational, and collective, as well as their contribution to change in number of hours worked, role overload, depression, and emotional exhaustion over time. We conducted a three-wave study with a four-month time separation between measurements among a sample of alumni from a French business school (*N* = 544) to explore these relationships. Results indicated that as expected, workaholism was positively associated with the individual self-concept, reflecting a tendency to prioritize individual achievements and success, but was also related to a stronger collective self-concept, which involves a self-definition based on group membership. Work engagement and affective organizational commitment were positively and only related to the collective self-concept. In terms of work outcomes, workaholism, but not work engagement and affective commitment, was found to increase the number of hours employees worked across time as well as to heighten their perception of being overloaded by their work. Workaholism was also associated with a significant increase in the odds of depression over time. In contrast, work engagement was found to protect employees from the risk of depression and emotional exhaustion over time. Affective commitment was unrelated to change in any of the four work outcomes. These findings have significant implications for research on heavy work investment and for our understanding of its nature and consequences for employees, which we elaborate on in the discussion.

## Workaholism, work engagement, and affective commitment: relationships to self-concept levels and work outcomes

As the boundaries between work and private life have been evanescing in the post-pandemic era as a result of remote working and other changes in labor organization (Hassard and Morris, 2022; Taris and de Jonge, 2024; Vyas, 2022), questions have arisen regarding the consequences of individuals’ work investment for their health and well-being (Gaudiino and Di Stefano, 2023; Spagnoli et al., 2020). The evolution of organizations may have rendered the frontier between healthy work investment and unreasonable devotion to work difficult to draw for numerous individuals. Moreover, research conducted in the pre-pandemic era has already established that heavy work investment may have both bright and dark sides for individuals (Harpaz and Snir, 2003; Snir and Harpaz, 2004, 2012). Therefore, distinguishing between healthy work investment and more detrimental forms of investment and examining their antecedents and consequences are particularly warranted in today’s world - where an estimated 14% of workers are affected by workaholism (Andersen et al., 2023).

Heavy work investment, a widely used notion that has an intuitive sense in folk theory, can be considered as an umbrella construct that subsumes different forms of psychological and behavioral investment into work and the larger workplace. Although the notion of heavy work investment has sometimes been equated with workaholism (e.g., Snir and Harpaz, 2012) and measured through the number of hours worked (e.g., Harpaz and Snir, 2003; Snir and Harpaz, 2004), the literature now widely acknowledges that heavy work investment encompasses distinct subtypes. For example, Snir and Harpaz (2012, 2021) identified four types of heavy workers: workaholics, work-devoted, organization-directed, and needy, primarily based on the controllability of their work investment. Rabenu and Shkoler (2022) took a different approach, characterizing different types of heavy workers based on their levels of both time and effort investment (e.g., Shkoler et al., 2017). We contend that all forms of heavy work investment are primarily characterized by an involvement of the self into one’s work activities, the work context, or the larger workplace. However, these forms may differ as to whether they include an affective, a cognitive, and/or a behavioral component and regarding the target of investment, which may be the activity of working in and of itself (Ng et al., 2007), one’s work (Macey and Schneider, 2008), or the larger workplace such as the organization (Meyer et al., 2004).

The present study focuses on three forms of heavy work investment, i.e., workaholism (Schaufeli et al., 2009), work engagement (Schaufeli et al., 2002; Schaufeli et al., 2008), and affective organizational commitment (hereafter affective commitment; Meyer and Allen, 1991; Meyer et al., 2004). There has been some disagreement about the conceptualization and measurement of workaholism, with some scholars considering the number of hours worked as a proxy for it (e.g., Snir and Harpaz, 2004). However, there is agreement from a conceptual perspective that workaholism involves (a) an addiction to work due to internal pressures, (b) “frequent thoughts about work when not working” and (c) putting hours into work beyond what is reasonable (Clark e al., 2016, p. 1840). This suggests that the definition of workaholism is broader than an excessive number of hours worked (Schaufeli et al., 2008). Relatedly, work engagement is defined “as a positive, fulfilling, work-related state of mind” composed of vigor, reflecting high levels of energy and resilience at work, dedication, referring to feelings of enthusiasm, pride, and challenge, and absorption, involving full concentration on one’s work (Schaufeli et al., 2002; Schaufeli et al., 2008). A similar conception of engagement has been proposed by Kahn (1990) who advocated that engagement reflects harnessing of the individual’s self with their work role through physical, emotional, and cognitive investments. This view has been operationalized through a measure of job engagement (Rich et al., 2010). While work engagement and job engagement share commonalities in conceptualization and measurement (Byrne et al., 2016), the engagement perspective from Kahn’s (1990) theory specifically includes an investment of the self into work (Macey and Schneider, 2008). We maintain that the investment of the self into the work role is central to engagement. Although workaholism and work engagement are conceptually distinct subtypes of heavy work investment, they are often described as opposite ends of a continuum (Tabak et al., 2021), with low engagement even being depicted as a feature of workaholism (Loscalzo and Giannini, 2017).Finally, the third construct of interest in this study is affective commitment, which has been defined as an employee’s emotional attachment to the organization (Meyer and Allen, 1991) based on an identification with its goals and values (Meyer et al., 2004; Vandenberghe et al., 2017). Because identity is at the core of affective commitment (i.e., individuals feel affectively committed when their goals and values match those of the organization), the self is involved in the process. Although affective commitment has been scarcely studied as a form of heavy work investment, it involves engaging in courses of action that are relevant to the organization (Meyer and Herscovitch, 2001), including citizenship behaviors. Such behaviors require time and effort beyond normal duties—the two core components of heavy work investment (Snir and Harpaz, 2012)—thereby increasing the risk of depletion and fatigue (Bolino et al., 2015). Empirical evidence indeed indicates that high levels of affective commitment may negatively impact employee health (Morin et al., 2013). These reasons suggest that affective commitment can be considered a relevant form of heavy work investment.

We posit that owing to their nature, workaholism, work engagement, and affective commitment should be associated with different drivers and outcomes, although some similarities in these relationships are likely to occur as the self is involved to some extent in either form of work investment. The first purpose of the present study is to associate the three types of heavy work investment with the levels of self-identity. Self-identities are knowledge structures that influence cognitive processing, reactions to external stimuli, and contain beliefs that people entertain about themselves, their social relationships, and their goals (Fiske and Taylor, 1991). These knowledge structures act as powerful self-regulatory mechanisms that influence work motivation, as well as the motives and goals that are pursued in the workplace (Cooper and Thatcher, 2010; Johnson et al., 2006, 2010). At the individual level, the self-concept is experienced as being separate from others, is based on a sense of uniqueness, and values the pursuit of individual achievements (Brewer and Gardner, 1996; Johnson et al., 2010). At the relational level, the self-concept prioritizes the definition of oneself in terms of relationships with specific others, and self-worth is viewed through the lens of meaningful dyadic relationships (Epitropaki et al., 2017; Johnson et al., 2010). Finally, at the collective level, the self-concept involves defining oneself in terms of group memberships where one’s self-worth is tied to the success and standing of the social group one belongs to (Johnson and Chang, 2006; Johnson et al., 2010). We argue that self-identity levels will be differentially linked to workaholism, work engagement, and affective commitment because the motives that drive behavior differ across self-concept levels (Cooper and Thatcher, 2010).

The second goal of the present study is to examine how the three forms of heavy work investment predict a series of work outcomes over time. Given the longstanding controversy concerning the extent to which workaholism (e.g., Balducci et al., 2018), work engagement (Mäkikangas et al., 2016), and affective commitment (e.g., Galais and Moser, 2009; Zheng et al., 2015) contribute to increased workload and reduced vs. improved well-being, we targeted outcomes that specifically tapped into these domains. Specifically, we examined the unique effects of the three forms of heavy work investment on change in the number of work hours, role overload, depression, and emotional exhaustion. For example, as the number of hours worked is a typical behavioral expression of workaholism (Clark et al., 2016; Schaufeli et al., 2008; Snir and Harpaz, 2012), one may expect workaholism to explain its increase over time. Relatedly, depression, which represents a syndrome characterized by depressed mood and loss of pleasure, has been namely associated with increased job strain (McTernan et al., 2013). Therefore, work engagement and possibly affective commitment, as psychological states imbued by positive affect and enjoyment, might be reflected in abundant resources that can reduce the odds of depression (Innstrand et al., 2012; Panaccio and Vandenberghe, 2012). In contrast, workaholism has been viewed as a state of addiction associated with a lack of pleasure (Balducci et al., 2018; Shimazu and Schaufeli, 2009), which may expose workaholics to a higher risk of depression (e.g., Yang et al., 2020). A similar pattern of relationships for the three forms of heavy work investment would be expected with emotional exhaustion, which expresses a less severe form of resource depletion compared to depression (Maslach et al., 2001).

This study contributes in several ways to the literature on heavy work investment. First, there is a dearth of research looking at the specific correlates and work-related and well-being outcomes of different forms of heavy work investment. Doing so would help clarify the unique properties of these forms, considering that the discussion on the bright and dark sides of workaholism and work engagement has been around for a while (e.g., Carse et al., 2017; Del Líbano et al., 2012), although it has not extended as much to affective commitment (e.g., Morin et al., 2013). Second, the present investigation counts among the first attempts to examine self-identity levels as correlates of the three targeted forms of heavy work investment. As self-identity levels determine the type of motive that underlies individuals’ attitudes, behavior, and goals in the workplace (Cooper and Thatcher, 2010), we expect significant relationships between some of the self-identity levels and the three forms of heavy work investment. Establishing these associations would be meaningful as this would help connect how information related to work tasks and the workplace is cognitively processed and how this processing could be explained by underlying motives for interactions with the environment. Third, our study examines the relative contribution of each of the three forms of heavy work investment to the intensity of work endeavors including the workload that ensues from such investment, as well as their consequences on ill-being. This approach is timely as it would feed the conversation on the relative benefits and drawbacks of different forms of heavy work investment. Finally, our study used a longitudinal design spanning over one year and involving three measurement times where a sample of alumni from a business school is tracked over time. Such sample is particularly suitable for exploring the correlates and consequences of heavy work investment, as managers and professionals (who represent a large portion of the sample) are known to invest significant time in, and experience strong attachment to, their work and workplace (Loi et al., 2018). Indeed, workers who are highly educated, hold managerial responsibilities, and work in white-collar jobs tend to be more prone to workaholism (Taris and de Jonge, 2024).

### Theoretical background and hypotheses

The antecedents of each of the three forms of heavy work investment have been explored to some extent, yet in separate areas of the literature. Women, and older and more educated individuals tend to display more workaholism (Taris and de Jonge, 2024). Personality factors such as perfectionism, neuroticism, and low conscientiousness (Balducci et al., 2018; Taris and de Jonge, 2024) are also associated with an increased risk of workaholism. Among work-related factors, workload (Balducci et al., 2020) and overwork culture (Afota et al., 2021) are known predictors of workaholism. Regarding work engagement, job resources (e.g., autonomy, social support, e.g., De Beer et al., 2020; Van Wingerden et al., 2021), personal resources (e.g., self-efficacy; Mazzetti et al., 2021a), job demands (e.g., Chong et al., 2020), transformational leadership (e.g., Pundt, 2021), and work-life balance (Brougham and Haar, 2021) are well-established predictors of the construct. Finally, perceived organizational support (Kurtessis et al., 2017), organizational justice (Colquitt et al., 2021), and transformational leadership (Ng, 2021) are among the main antecedents of affective organizational commitment.

The present study aims to extend this previous research by examining the joint influence of self-identity levels on each of the three forms of heavy work investment. The following section elaborates on how self-identity levels—and which specific levels—may contribute to workaholism, work engagement, and affective commitment. The subsequent section explores how these forms of heavy work investment may relate differently to work outcomes, specifically the number of hours worked, role overload, depression, and emotional exhaustion.

### Specific hypotheses on self-identity levels and forms of heavy work investment

The self-concept is a knowledge structure about the self where information, beliefs, and perceptions about oneself and others are stored. This knowledge structure influences individuals’ cognition, attitudes, and behavior (Fiske and Taylor, 1991; Lord and Brown, 2004; Markus and Wurf, 1987). Research has identified three levels of self-concept. The individual self-concept reflects a self-conception based on a sense of individuation, where self-worth is derived from personal achievements, career success, and recognition at work (Johnson et al., 2006). Self-enhancement is the basic motive associated with the individual self-concept. This motive implies a concern for gaining prestige for one’s own accomplishments (Cooper and Thatcher, 2010; Zhang and Alicke, 2021). Moreover, self-enhancement is accompanied by “a desire for continuity in the self across time and across personal attributes” (Cooper and Thatcher, 2010, p. 524), which represents a need for self-consistency. The relational self-concept reflects a self-definition based on dyadic relationships, where self-worth is derived from entertaining satisfying relationships with significant others (Robert and Vandenberghe, 2021). In this level of the self-concept, individuals feel prompted to reduce the uncertainty surrounding their dyadic relationships by investing time to make them work (Cooper and Thatcher, 2010). Finally, the collective self-concept primarily relates to individuals seeing themselves as members of social groups (Johnson and Chang, 2006). Such self-concept leads individuals to feel concerned about the welfare of their group (Brewer and Gardner, 1996; Steffens et al., 2021). In this level, individuals’ attachment to the group is experienced through a depersonalized belongingness motive, which refers to a perceived prototypical similarity to others (van Knippenberg et al., 2004).

We first suggest that among self-concept levels, the individual self-concept is likely the most salient driver of workaholism. Historical work on workaholism has indicated that a core aspect of the construct is “an addiction to work, the compulsion or uncontrollable need to work incessantly” (Oates, 1971, p. 11). Spence and Robbins (1992) later posited that the workaholic is (a) highly invested in their work, (b) feels driven to work as a result of internal pressures, and (c) experiences little enjoyment in working. More recently, Schaufeli et al. (2008) conceptualized workaholism as subsuming two components: (a) a behavioral component reflecting a tendency to work excessively and beyond the normal expectations set by an employer (which is a reason why the number of worked hours has been sometimes used to measure the construct; Clark et al., 2016), and (b) a cognitive component characterized by an inner drive that makes the workaholic constantly thinking about their work and feeling guilty when not working (Schaufeli et al., 2009). This approach has resulted in the most widely validated measure of workaholism to date comprising two five-item scales (working excessively and working compulsively) (Dutch Workaholism Scale; Schaufeli et al., 2009). Given this conceptualization of workaholism, it seems obvious that workaholism has a close connection to the individual self-concept. For example, workaholics feel the need for themselves to work intensely, suggesting that the activity of working contributes to defining their identity as unique individuals (Ng et al., 2007). Moreover, the intensity of the investment into working means that the notion of work accomplishments and recognition are prominent sources of motivation. Such a source of motivation is central to the individual self-concept (Tang and Vandenberghe, 2022). Moreover, from the perspective of social identity theory (Tajfel and Turner, 1979), self-worth and esteem act as drivers that tie individuals’ professional achievements and recognition to their social group (i.e., the organization). One may thus expect workaholism to represent a means through which people with an individual self-concept gain recognition (i.e., a sense of self-worth and esteem) within the organization. Therefore, we propose the following hypothesis.

*H1:* Among the self-concept levels, the individual self-concept will be uniquely and positively associated with workaholism.

Work engagement might be related to both the individual self-concept and the collective self-concept. For example, Schaufeli et al. (2002) defined work engagement as comprising dimensions that are opposite to those of burnout, i.e., vigor, dedication, and absorption. These dimensions include energetic and emotional investments into work activities, which might be associated with the individual self-concept. Indeed, without energy and emotional involvement, it might be difficult for an individualist to achieve their work to the standards they set for themselves (i.e., success and recognition) (Johnson et al., 2010). Besides a connection to the individual self-concept, work engagement may also be related to the collective self-concept. While examining the deep nature of engagement, Macey and Schneider (2008) noted that “engagement as a state has a strong affective tone connoting, at a minimum, high levels of involvement (passion and absorption) in the work and the organization (pride and identity) as well as affective energy (enthusiasm and alertness) and a sense of self-presence in the work,” which reflects the harnessing of the self with the work role (Kahn, 1990). However, given the importance of the affective tone underlying engagement, the construct shares commonalities with other constructs such as job satisfaction and affective commitment (Macey and Schneider, 2008). Following this view, engagement may include an attachment that is broader than the work itself. Just as positive affectivity can foster belongingness to social groups (Li et al., 2019; Vandenberghe et al., 2019), it is likely that work engagement, thanks to its affective basis, may facilitate social integration in the workplace. Indeed, from a social identity theory perspective (Tajfel and Turner, 1979), such social integration helps achieve social categorization within the organization, thereby reinforcing the link with the organization. Thus, the collective self-identity level, due to its associated desire to belong to social groups, may be positively associated with work engagement. The above reasoning leads to the following hypothesis.

*H2:* Among the self-concept levels, the individual self-concept (Hypothesis 2a) and the collective self-concept (Hypothesis 2b) will be uniquely and positively associated with work engagement.

Finally, affective commitment involves the identification with the goals and values of the organization (Meyer and Allen, 1991; Meyer et al., 2004; Meyer and Herscovitch, 2001). As employees with high levels of affective commitment come to define themselves in terms of the characteristics of the organization, they tend to view themselves in similar terms as the organization. This suggests that such employees feel a sense of belonging to the organization as a social group. There is thus a clear commonality between affective commitment and collective self-identity, which involves sensitivity and attraction to the norms and goals of the social groups to which individuals belong (Brewer and Gardner, 1996). Moreover, the source of motivation behind the collective self-concept is a sense of contributing to the welfare of collective entities. It is also evident from a social identity theory perspective that affective commitment involves a self-conception where self-categorizing oneself as a member of the organization builds self-worth (Steffens et al., 2021). Supporting this view, Johnson and Chang (2006) found the collective self-concept to be positively related to affective commitment. We thus propose the following hypothesis as a constructive replication.

*H3:* Among the self-concept levels, the collective self-concept will be uniquely and positively associated with affective commitment.

### Specific hypotheses on forms of heavy work investment and work outcomes

Past theorizing on workaholism, work engagement, and affective commitment has implicitly or explicitly alluded to the idea that all three forms of heavy work investment may contribute to an increase in the number of hours people devote to working. But do they really do? Although the approach has been rather abandoned today, workaholism has been measured by some researchers through the number of hours worked (e.g., Harpaz and Snir, 2003; Snir and Harpaz, 2004). There is now agreement among scholars that the number of hours provides an incomplete picture of workaholism, essentially because such a measure does not consider the reasons why workaholics tend to work more hours than others (Clark et al., 2016; Ng et al., 2007). Nonetheless, despite the imperfection of this measure, a positive relationship between workaholism and the number of work hours is expected because the latter remains a behavioral expression of workaholism. In contrast, it is less clear that work engagement and affective commitment would be uniquely related to the number of work hours. On one hand, work engagement is built from energetic resources, resilience, persistence at work, and enthusiasm (Macey and Schneider, 2008; Schaufeli et al., 2002), all of which may indirectly predispose individuals to put more hours into work. However, at the same time, such states may involve quality work where passion and meaning are part of the process. These notions may not imply a high number of hours worked. Therefore, work engagement may not be predictive of more hours worked incrementally to workaholism. Similarly, affective commitment has been thought of as involving extra effort at work. For example, early on, Mowday et al. (1979, p. 226) argued that one of the three factors characterizing organizational commitment was “a willingness to exert considerable effort on behalf of the organization.” This would suggest a link to long hours worked. However, again, the construct of effort is a complex notion that is generally ill-defined (Macey and Schneider, 2008). Thus, effort may not necessarily lead to an increase in the number of hours worked. Accordingly, we would not expect affective commitment to predict the number of work hours beyond the influence of workaholism. To summarize, the following hypothesis is proposed.

*H4:* Among the three forms of heavy work investment, workaholism will uniquely predict an increase in the number of work hours over time.

Role overload, a work stressor representing a perceived imbalance between role demands and employees’ resources such as time, skills, and energy (Eatough et al., 2011; Rizzo et al., 1970) is costly to employees and organizations (Alfes et al., 2018). Role overload is associated with increased psychological strain (Glazer and Beehr, 2005; Örtqvist and Wincent, 2006) and less organizational citizenship behavior (Eatough et al., 2011) and job performance (Gilboa et al., 2008). Among the three forms of heavy work investment, workaholism appears as the most likely to foster an increase in role overload over time. Two reasons may support this prediction. First, part of the essence of workaholism involves an addiction to work, suggesting that workaholics may voluntarily seek more work responsibilities than is normally expected from the employment contract (Ng et al., 2007). This process may end up overwhelming them with more workload than they can manage, creating conditions for enhanced role overload. Second, as Snir and Harpaz (2012) suggested, workaholics may consciously seek to join organizations or select jobs where job demands are high, which would be in line with the high standards of achievement they set for themselves (see also Clark et al., 2016). Regarding work engagement and affective commitment, their influence on role overload is less clear. On one hand, the enthusiasm and energy associated with work engagement make it a potential predictor of increased workload. However, work engagement is not an addiction to work but rather an investment into work based on the sense of meaning that it brings to the individual’s life. Such investment corresponds to workload that has a qualitative component, which differs from role overload. Similarly, individuals with high levels of affective commitment are inclined to have a broader definition of their job responsibilities (Morrison, 1994; Tang and Vandenberghe, 2020). Yet such broadening of work responsibilities may not mean experiencing an imbalance between job demands and personal resources. Thus, the link between affective commitment and role overload over time is not evident. To sum up, we posit that workaholism is likely the most relevant predictor of increased role overload over time.

*H5:* Among the three forms of heavy work investment, workaholism will uniquely predict an increase in role overload over time.

Finally, we expect the three forms of heavy work investment to exert meaningful effects on well-being outcomes, particularly depression and emotional exhaustion. First, there is agreement among scholars that workaholism involves an addiction to work driven by internal pressures that are associated with low levels of enjoyment at work (Clark et al., 2016; Schaufeli et al., 2009; Spence and Robbins, 1992). Moreover, research indicates that workaholism is associated with higher job-related negative affect (Balducci et al., 2018). Meta-analytic evidence also shows that workaholism is related to stronger trait and state negative affect (Clark et al., 2016). As both depression, which reflects a disorder characterized by feelings of sadness, absence of pleasure, sleep problems, and loss of interest in activities (McTernan et al., 2013; Ryu et al., 2024), and emotional exhaustion, characterized by a sense of depletion of one’s emotional resources (Maslach et al., 2001), are deeply related to some affective disorder, these states should be accentuated by workaholism. Second, work engagement, conceptualized as the opposite of burnout (Maslach et al., 2001), i.e., as a state of well-being characterized by a sense of energetic and efficacious connection with work activities (Schaufeli et al., 2002), should involve a reservoir of emotional resources that helps protect individuals from emotional exhaustion. Third, research evidence indicates that affective commitment is negatively associated with emotional exhaustion (e.g., Tang and Vandenberghe, 2020), which can be explained by the fact that affectively committed employees experience a sense of self-consistency when working at the organization owing to a fit between personal values and organizational values (Meyer et al., 2004). To summarize, we contend that all three forms of heavy work investment should contribute to depression and emotional exhaustion over time, although in different ways. Thus, the following hypothesis is proposed.

*H6:* Among the three forms of heavy work investment, workaholism will uniquely predict an increase in depression and emotional exhaustion over time (Hypothesis 6a), while work engagement and affective commitment will uniquely predict a decrease in depression and emotional exhaustion over time (Hypothesis 6b).

## Materials and methods

### Sample and procedure

As part of a larger project, we surveyed the alumni of a French business school at three points in time, using a four-month time separation between waves. The data was originally collected in 2017 from alumni of the Master program who graduated between 2011 and 2015. At each wave, participants received an email invitation to complete surveys. Participation was encouraged by offering respondents the possibility of making a $5 gift to a Charity selected among six options. Only respondents who had salaried employment were retained. Participants were informed of the objectives of the research and ensured that responses would remain confidential. They could answer a French or English version of the surveys. A four-month time lag was chosen due to its relevance in examining the longer-term health effects of workaholism (Taris and de Jonge, 2024). Indeed, longer time intervals allow for the observation of true changes over time (Zhao et al., 2024), which is particularly important considering that emotional exhaustion and depressive symptoms can take time to develop (Schermuly and Meyer, 2016). Moreover, using a time lag shorter than one year aligns with experts’ recommendations (Dormann and Griffin, 2015) and reduces the likelihood that confounding variables will affect the observed effects (Zhao et al., 2024).

At Time 1, we measured workaholism, work engagement, affective commitment, the three levels of the self-concept, and demographics. At Time 2, we measured the number of work hours, role overload, depression, and emotional exhaustion (all self-reported), which served as baseline controls in the analyses predicting work outcomes. At Time 3, we measured again the number of work hours, role overload, depression, and emotional exhaustion which were used as dependent variables of the forms of heavy work investment.

At Time 1, usable responses were obtained from 544 participants, while 266 responses were obtained at Time 2, and 181 at Time 3, representing a 33% response rate from Time 1 data. At Time 1, average age was 37.55 years (*SD* = 8.97), average organizational tenure was 5.11 years (*SD* = 5.48), and 53% of the respondents were women. Respondents mainly worked full-time (96%) and most of them completed the French version of the questionnaires (97%). In the sample, 37% were professionals or specialists with no supervisory responsibilities while 62% were managers, mid-level managers, or upper-level managers. Most of the respondents (97.3%) held a Master’s degree. In terms of organization size, the sample was distributed as follows: < 100 employees: 18%; 100–1,000 employees: 23%; > 1,000 employees: 59%. Various industries were represented in the sample such as banking and insurance (16%), professional, scientific, and technical services (11%), manufacturing (6%), and retail (5%).

### Measures

All scale items (except the number of work hours) were rated using a 5-point scale, ranging from 1 (*strongly disagree*) to 5 (*strongly agree*), except those related to workaholism. A 5-point frequency scale was used for workaholism, which ranged from 1 (*never*) to 5 (*often, nearly every day*). French versions of English scales were developed following a translation-back-translation procedure (Schaffer and Riordan, 2003).

#### Workaholism (Time 1)

We used the 10-item Dutch Work Addiction Scale (DUWAS; Schaufeli et al., 2009) to measure workaholism. While this scale comprises two 5-item dimensions, namely working compulsively (e.g., “I feel that there is something inside me that drives me to work hard”) and working excessively (e.g., “I seem to be in a hurry and racing against the clock”), it is common to combine scores on the two dimensions to create an overall score of workaholism (e.g., Balducci et al., 2018; Clark et al., 2016). The reliability of this scale was 0.85 in this study.

#### Work engagement (Time 1)

We measured work engagement through the 9-item version of the Utrecht Work Engagement Scale (UWES; Schaufeli and Bakker, 2003), which comprises three items for vigor, dedication, and absorption, respectively. A typical item is “At my job, I feel strong and vigorous” (vigor dimension). We used a single score on work engagement by averaging scores across the three factors because they were highly correlated with one another. The reliability for this scale was 0.90 in this study.

#### Affective commitment (Time 1)

The 6-item version of the affective commitment scale developed by Meyer et al. (1993) and further adapted by Vandenberghe et al. (2007) was used in this study. A sample item is “I really feel that I belong in this organization.” The reliability for this scale was 0.93 in this study.

#### Self-concept levels (Time 1)

Selenta and Lord’s (2005) Levels of the Self-Concept Scale (LSCS) (see also Johnson et al., 2006) was used to measure the three self-concept constructs. The individual (e.g., “I feel best about myself when I perform better than others”), relational (e.g., “It is important to me that I uphold my commitments to significant people in my life”), and collective (e.g., “feel great pride when my team or group does well, even if I am not the main reason for its success”) self-concepts were each measured through five items. Reliabilities for these scales were 0.82, 0.68, and 0.76, respectively. Note that while no hypothesis was associated with the relational self-concept, it is common to include it when testing the effect of the other two levels because they tend to be moderately correlated (Robert and Vandenberghe, 2021).

#### Number of work hours (Time 2 and Time 3)

Respondents were invited to report the total number of hours they worked on average per week including those at the office and outside the office. On average, respondents reported working 49.11 h per week (*SD* = 10.19) at Time 2 and 49.22 h per week (*SD* = 9.90) at Time 3.

#### Role overload (Time 2 and Time 3)

We used Schaubroeck et al.’s (1989) three-item scale to measure role overload. A sample item is “It often seems like I have too much work for one person to do.” The alpha coefficient for this scale was 0.93 in this study at Time 2 and 0.94 at Time 3.

#### Depression (Time 2 and Time 3)

Depression was measured using a scale developed by Salokangas et al. (1994) and further validated by Vuori and Vinokur (2005). Respondents reported the extent to which they were affected by a series of depressive symptoms during the past month. Typical symptoms included “feeling blue,” and “not enjoying life.” Note that the item “sleeping disorders,” which reflects a somatic instead of a psychological complaint was dropped, thereby reducing the scale to 9 items. The alpha coefficients for this scale were 0.92 and 0.93 at Time 2 and Time 3, respectively.

#### Emotional exhaustion (Time 2 and Time 3)

A five-item version (e.g., Lapointe et al., 2011) of Schaufeli et al.’s (1996) Maslach Burnout Inventory–General Survey (MBI-GS) was used to assess emotional exhaustion. A sample item is “I feel emotionally drained from my work.” The reliability for this scale was 0.88 at Time 2 and 0.90 at Time 3.

#### Control variables

To render our analyses predicting work outcomes at Time 3 more robust, we controlled for the baseline levels of the outcomes at Time 2. This allowed testing whether the three forms of heavy work investment predicted change in the outcomes between Time 2 and Time 3. As our analyses predicting work outcomes at Time 3 controlled for their baseline level at Time 2, it was not necessary to control for other variables such as demographics in these analyses (Zapf et al., 1996). However, when testing the relationships between self-concept levels and the three forms of heavy work investment, demographics (i.e., age, gender, organizational tenure, education level, job level, employment status, and organization size) were included as controls.

## Results

### Confirmatory factor analyses

We first tested the dimensionality of our multi-item scales by conducting a confirmatory factor analysis (CFA) through Mplus 8.6 (Muthén and Muthén, 2010) with maximum likelihood (ML) estimation. To simplify the measurement model, we used a parceling approach (Little et al., 2013) by randomly aggregating items to form three indicators per construct (i.e., Time 1 affective commitment, and self-concept levels, and Time 3 role overload, depression, and emotional exhaustion). The items of Time 1 work engagement and workaholism were grouped into three and two indicators, respectively, according to their theoretical dimensions. Standardized factor loadings for the indicators ranged from 0.60 to 0.94, with an average of 0.82, demonstrating strong convergent validity for our constructs (Cheung et al., 2024). Results of the CFAs are presented in Table 1. The hypothesized 9-factor model exhibited a reasonably good fit to the data, *χ*^2^(263) = 583.89, *p* < 0.001, CFI = 0.95, TLI = 0.94, RMSEA = 0.047, SRMR = 0.053, and yielded a better fit than any alternative model obtained by combining factors on a two-by-two basis (*p* < 0.001). Interestingly, the results showed that our hypothesized model fit the data better than a model combining workaholism, work engagement, and affective commitment [*Δ χ*^2^(15) = 1017.99, *p* < 0.001]. These results suggest that our variables were distinguishable. Moreover, correlations between the three types of heavy work investment were lower than 0.60, which further supports the discriminant validity of these variables (Cheung et al., 2024).

**Table 1:** Fit indices for confirmatory factor analysis models.

Model	*χ* ^2^	*df*	Δ *χ*^2^	Δ *df*	CFI	TLI	RMSEA	SRMR
1.Theorized nine-factor model	583.89*	263	-	-	0.95	0.94	0.047	0.053
2.Combining workaholism and work engagement	993.80*	271	409.91*	8	0.88	0.86	0.099	0.070
3.Combining workaholism and affective commitment	997.24*	271	413.35*	8	0.88	0.86	0.070	0.096
4.Combining work engagement and affective commitment	1199.66*	271	615.77*	8	0.85	0.82	0.079	0.081
5.Combining workaholism and role overload	808.27*	271	224.38*	8	0.91	0.89	0.060	0.081
6.Combining workaholism and depression	894.18*	271	310.29*	8	0.90	0.88	0.065	0.127
7.Combining workaholism and emotional exhaustion	902.76*	271	318.87*	8	0.90	0.88	0.065	0.137
8.Combining work engagement and role overload	1139.24*	271	526.82*	8	0.86	0.83	0.077	0.126
9.Combining work engagement and depression	1110.71*	271	436.57*	8	0.86	0.84	0.075	0.117
10.Combining work engagement and emotional exhaustion	1020.46*	271	554.78*	8	0.88	0.85	0.071	0.119
11.Combining affective commitment and role overload	1138.67*	271	646.39*	8	0.86	0.83	0.077	0.126
12.Combining affective commitment and depression	1230.28*	271	551.28*	8	0.84	0.81	0.081	0.148
13.Combining affective commitment and emotional exhaustion	1135.17*	271	409.91*	8	0.86	0.83	0.077	0.149
14.Combining workaholism, work engagement and affective commitment	1601.88*	278	1017.99*	15	0.78	0.75	0.094	0.111

N = 544, based on full information maximum likelihood estimation. CFI, comparative fit index; TLI, Tucker-Lewis index; RMSEA, root-mean-square error of approximation; SRMR, standardized root mean square residual.*p < 0.001.

### Attrition analyses

Attrition analyses were conducted to check whether the probability of remaining in the sample at Time 2 and Time 3 was influenced by demographics (i.e., age, gender, organizational tenure) and Time 1 substantive variables (i.e., workaholism, affective commitment, and work engagement). Logistic regression results showed that the model did not significantly predict the probability of dropping from the sample between Time 1 and Time 2 [*χ*^2^(6) = 9.47, *ns*] and between Time 2 and Time 3 [*χ*^2^(6) = 9.05, *ns*]. However, work engagement significantly predicted attrition between Time 1 and Time 2 (*b* = 0.33, *p* < 0*.05*). As Hypotheses 4 to 6 involved predicting change in the outcomes between Time 2 and Time 3, the attrition attributable to work engagement between Time 1 and Time 2 does not threaten the validity of our analyses.

### Descriptive statistics and correlations

Table 2 displays the descriptive statistics and correlations for the study variables. Interestingly, while the collective self-concept positively correlates with workaholism (*r* = 0.24, *p* < 0.01), work engagement (*r* = 0.31, *p* < 0.01), and affective commitment (*r* = 0.40, *p* < 0.01), the individual self-concept is only significantly (positively) related to workaholism (*r* = 0.21, *p* < 0.01). Moreover, workaholism correlates positively with Time 3 number of work hours (*r* = 0.36, *p* < 0.01), role overload (*r* = 0.40, *p* < 0.01), depression (*r* = 0.33, *p* < 0.01), and emotional exhaustion (*r* = 0.27, *p* < 0.01). In contrast, work engagement is unrelated to Time 3 number of work hours (*r* = 0.12, *ns*) and role overload (*r* = 0.02, *ns*), but is negatively related to depression (*r* = −0.36, *p* < 0.01) and emotional exhaustion (*r* = −0.38, *p* < 0.01). Similarly, affective commitment is unrelated to Time 3 number of work hours (*r* = 0.06, *ns*) and role overload (*r* = 0.05, *ns*), but is negatively related to depression (*r* = −0.15, *p* < 0.05) and emotional exhaustion (*r* = −0.15, *p* < 0.05).

**Table 2:** Means, standard deviations, and correlations among variables.

Variable	*M*	*SD*	1	2	3	4	5	6	7	8	9	10	11	12	13	14	15	16	17	18	19	20	21
1.Age (years)	37.55	8.97	–																				
2.Gender	1.53	0.50	−0.17**	–																			
3.Organizational tenure (years)	5.11	5.48	0.42**	−0.11*	–																		
4.Education level	3.97	0.19	−0.01	0.05	−0.06	–																	
5.Job level	3.09	1.11	0.44**	−0.15**	0.17**	0.03	–																
6.Employment status	1.04	0.19	0.08	0.14**	0.06	0.03	−0.11*	–															
7.Organization size	5.00	1.71	−0.03	−0.07	0.09*	0.05	0.01	−0.14**	–														
8.Individual self-concept (T1)	2.92	0.92	−0.24**	−0.13**	−0.08	0.06	−0.07	−0.08	0.05	(0.82)													
9.Relational self-concept (T1)	4.42	0.50	−0.06	0.10*	−0.08	0.04	−0.06	0.02	−0.01	0.09	(0.68)												
10.Collective self-concept (T1)	4.17	0.61	0.13**	0.04	−0.02	0.03	0.07	−0.01	0.02	0.09*	0.29**	(0.76)											
11.Work engagement (T1)	3.40	0.80	0.13**	−0.02	0.06	−0.00	0.20**	−0.01	0.03	0.02	0.05	0.31**	(0.90)										
12.Affective commitment (T1)	3.22	1.02	0.07	−0.07	0.11**	−0.09	0.14**	0.05	−0.02	0.04	0.07	0.40**	0.51**	(0.93)									
13.Workaholism (T1)	3.51	0.74	0.08	0.02	0.03	−0.02	0.21**	−0.11*	0.07	0.21**	0.14**	0.24**	0.22**	0.17**	(0.85)								
14.Number of work hours (T2)	49.11	10.19	0.15*	−0.13*	−0.06	0.08	0.40**	−0.20**	0.11	0.01	0.03	0.10	0.20**	0.11	0.43**	–							
15.Role overload (T2)	3.34	1.17	0.05	0.05	0.00	0.04	0.17**	−0.07	−0.08	−0.01	0.07	0.02	0.03	−0.01	0.42**	0.36**	(0.93)						
16.Depression (T2)	2.59	1.01	−0.14*	0.16*	−0.02	0.02	−0.06	0.03	−0.00	0.17**	0.18**	−0.02	−0.46**	−0.26**	0.21**	−0.01	0.18**	(0.92)					
17.Emotional exhaustion (T2)	2.71	1.13	−0.13*	0.17**	−0.02	0.00	0.04	0.01	−0.01	0.09	0.09	−0.06	−0.40**	−0.19**	0.33*	0.11	0.40**	0.79**	(0.88)				
18.Number of work hours (T3)	49.22	9.90	0.17*	−0.19*	0.09	0.13	0.37**	−0.13	0.15*	0.14	0.10	0.19**	0.12	0.06	0.36**	0.69**	0.28**	0.01	0.08	–			
19.Role overload (T3)	3.39	1.12	−0.09	0.06	−0.07	0.11	0.23**	0.00	−0.03	0.14	0.20**	0.05	0.02	0.05	0.40**	0.30**	0.72**	0.30**	0.43**	0.35**	(0.94)		
20.Depression (T3)	2.61	1.04	−0.12	0.12	−0.08	0.01	−0.03	0.01	−0.07	0.17*	0.10	0.03	−0.36**	−0.15*	0.33**	0.03	0.26**	0.67**	0.62**	−0.03	0.31**	(0.93)	
21.Emotional exhaustion (T3)	2.68	1.12	−0.14	0.10	−0.08	0.04	−0.03	0.05	−0.06	0.10	0.09	0.00	−0.38**	−0.15*	0.27**	0.01	0.39**	0.58**	0.66**	0.01	0.46**	0.79**	(0.90)

Ns = 544–181. T1 = Time 1; T2 = Time 2. For Gender: 1 = male, 2 = female; for Education level: 1 = high school, 2 = college; 3 = undergraduate, 4 = master’s or doctorate; for Job level: 1 = intern or novice, 2 = analyst or professional, 3 = manager of a small team, 4 = manager of a team with more than 5 employees, 5 = director, vice-president, or equivalent; for Employment status: 1 = full-time, 2 = part-time; for Organization size: 1 = ≤20 employees, 2 = 21–50 employees, 3 = 51–100 employees, 4 = 101–300 employees, 5 = 301–500 employees, 6 = 501–1,000 employees, 7 = 1,000+ employees. Reliability coefficients are reported in parentheses along the diagonal.

*p < 0.05; **p < 0.01.

### Hypothesis testing

We examined our hypotheses through multiple regression analyses using SPSS (version 26). We first examined the contributions of the self-concept levels to each form of heavy work investment. The results are presented in Table 3. We entered demographic variables in the first step and introduced the self-concept levels in the second step. Interestingly, job level was the sole demographic variable positively predicting workaholism (*β* = 0.21, *p* < 0.001), work engagement (*β* = 0.18, *p* < 0.001), and affective commitment (*β* = 0.16, *p* < 0.01) (Table 3, Model 1 s). As predicted in Hypothesis 1, Table 3 (Model 2) indicates that the individual self-concept was positively related to workaholism (*β* = 0.22, *p* < 0.001). However, though not anticipated, the collective self-concept was also positively related to workaholism (*β* = 0.15, *p* < 0.001). Relatedly, the collective self-concept was positively associated with work engagement (*β* = 0.30, *p* < 0.001) while the individual self-concept was not (*β* = 0.01, *ns*) (Table 3, Model 2). Thus, Hypothesis 2b is supported while Hypothesis 2a is rejected. Finally, the collective self-concept was positively related to affective commitment (*β* = 0.41, *p* < 0.001) (Table 3, Model 2), hence Hypothesis 3 is supported.

**Table 3:** Results of linear regression analyses for work engagement, affective commitment, and workaholism.

		Work engagement		Affective commitment		Workaholism	
Step	Variable(s) entered	Model 1	Model 2	Model 1	Model 2	Model 1	Model 2
1	Age	0.05	0.00	−0.06	−0.13*	0.00	0.04
	Gender	0.02	0.00	−0.05	−0.07	0.06	0.08
	Organizational tenure	0.00	0.03	0.10*	0.13**	−0.00	0.01
	Education level	−0.01	−0.02	−0.08	−0.09*	−0.03	−0.05
	Job level	0.18***	0.17***	0.16**	0.14***	0.21***	0.21***
	Employment status	0.01	0.02	0.07	0.08*	−0.08	−0.07
	Organization size	0.03	0.02	−0.02	−0.04	0.07	0.05
	Δ*R*^2^	0.04**		0.05***		0.06***	
2	Individual self-concept		0.01		0.00		0.22***
	Relational self-concept		−0.03		−0.04		0.08
	Collective self-concept		0.30***		0.41***		0.15***
	Δ*R*^2^		0.08***		0.15***		0.09***

Except for Δ*R*^2^ rows, entries are standardized regression coefficients. Final model statistics: Work engagement: *F* (10, 519) = 7.31, *p* < 0.001, *R*^2^ = 0.13; Affective commitment: *F* (10, 519) = 12.65, *p* < 0.001, *R*^2^ = 0.20; Workaholism: *F* (10, 519) = 8.64, *p* < 0.001, *R*^2^ = 0.15.

*p < 0.05; **p < 0.01; ***p < 0.001.

Hypotheses 4–6 pertained to the contribution of each form of heavy work investment to the selected work outcomes. The results are presented in Table 4. In all models, the baseline level of the outcome at the preceding time was controlled for in the analysis. As can be seen from Table 4 (Model 2 s), workaholism contributed to a significant increase in both number of work hours (*β* = 0.14, *p* < 0.05) and role overload (*β* = 0.14, *p* < 0.05) over time. Hypotheses 4 and 5, respectively, are thus supported. Furthermore, workaholism contributed to an increase in the likelihood of depression (*β* = 0.18, *p* < 0.01) but not emotional exhaustion (*β* = 0.06, *ns*) over time (Table 4, Model 2 s). Hypothesis 6a is thus partly supported. Relatedly, work engagement was significantly related to a decrease over time in depression (*β* = −0.17, *p* < 0.05) and emotional exhaustion (*β* = −0.16, *p* < 0.05) (Table 4, Model 2 s). In contrast, affective commitment was unrelated to change over time in depression (*β* = 0.02, *ns*) and emotional exhaustion (*β* = 0.00, *ns*) (Table 4, Model 2 s). Hypothesis 6b is thus partly supported.

**Table 4:** Results of linear regression analyses for Time 3 number of work hours, role overload, depression, and emotional exhaustion.

		T3 Number of work hours		T3 Role overload		T3 Depression		T3 Emotional exhaustion	
Step	Variable(s) entered	Model 1	Model 2	Model 1	Model 2	Model 1	Model 2	Model 1	Model 2
1	T2 Number of work hours	0.68***	0.62***						
	T2 Role overload			0.72***	0.66***				
	T2 Depression					0.66***	0.53***		
	T2 Emotional exhaustion							0.66***	0.57***
	Δ*R*^2^	0.46***		0.51***		0.43***		0.43***	
2	T1 Work engagement		0.01		0.01		−0.17*		−0.16*
2	T1 Affective commitment		0.01		0.05		0.02		0.00
2	T1 Workaholism		0.14*		0.14*		0.18**		0.06
	Δ*R*^2^		0.02		0.02		0.04*		0.02

T1 = Time 1; T2 = Time 2; T3 = Time 3. Except for Δ*R*^2^ rows, entries are standardized regression coefficients. Final model statistics: T3 Number of work hours: *F* (4, 175) = 38.46, *p* < 0.001, *R*^2^ = 0.47; T3 Role overload: *F* (4, 175) = 48.86, *p* < 0.001, *R*^2^ = 0.53; T3 Depression: *F* (4, 175) = 37.54, *p* < 0.001, *R*^2^ = 0.47; T3 Emotional exhaustion: *F* (4, 175) = 34.72, *p* < 0.001, *R*^2^ = 0.45.

**p < 0.01; ***p < 0.001.

## Discussion

In this study, using a sample of 544 employees, we investigated the associations between the three adjacent constructs of workaholism, work engagement, and affective commitment with levels of self-concept (i.e., individual, relational, and collective). We then examined their predictive effects on changes in work hours, role overload, depression, and emotional exhaustion over time (i.e., eight months later). The findings reveal that, while collective self-concept correlates with all three types of heavy work investment, individual self-concept is only associated with workaholism. We further found that workaholism predicts increases in self-reported work hours and role overload eight months later, as well as elevated levels of depression (though not emotional exhaustion). In contrast, work engagement was found to predict a reduction in levels of depression and of emotional exhaustion but was unrelated to changes in work hours and role overload. Affective commitment was unrelated to any of the four studied outcomes.

### Theoretical implications

These findings corroborate the interconnected yet distinctive nature of workaholism, work engagement, and affective commitment. Distinguishing between heavy work investment types is critical to prevent construct proliferation (Shaffer et al., 2016) and to recognize different work psychological states that produce contrasting employee and organizational outcomes (Snir and Harpaz, 2012). While the three constructs showed significant positive correlations – particularly strong (*r* = 0.51, *p* < 0.01) between work engagement and affective commitment – they each exhibited distinct nomological networks. This study thus adds to a growing body of work (e.g., Bereznowski et al., 2023; Di Stefano and Gaudiino, 2019; Shimazu et al., 2014) examining patterns of differences between workaholism and work engagement, constructs that remain conceptually hard to discern (Di Stefano and Gaudiino, 2019). We also contribute to establishing their distinctiveness with affective commitment, a conceptually related construct (Hallberg and Schaufeli, 2006). This approach has rarely been taken in the field, where most research has treated affective commitment as an outcome - rather than as an adjacent construct - of workaholism and work engagement.

Interestingly, although all three forms of work investment were correlated with a collective self-orientation, only workaholism was linked to an individual self-orientation. This finding is consistent with prior research that has established correlations between achievement striving, ambition, and workaholism (Clark et al., 2016). An individual self-concept typically manifests through self-enhancement motives (Cooper and Thatcher, 2010), commonly associated with values, such as ambition, competitiveness, and achievement, which are known to drive workaholism (Clark et al., 2016). However, contrary to our predictions, work engagement was not associated with a higher individual self-concept. This finding contrasts with cross-cultural research suggesting that organizational and country cultures that promote individual accomplishments, competencies, and self-fulfillment generally enhance work engagement (e.g., Hu et al., 2014). However, at the individual level, an individual orientation tends to predict self-serving goals, which does not align with the conceptualization of work engagement. For example, Johnson and Chang (2006) observed that employees with strong individual self-concepts have stronger continuance commitment, where ties to the organization are rooted in the costs associated with leaving it (Meyer and Allen, 1991). Such calculation-based investment does not correspond with the definition of employee work engagement, where pleasure and immersion in work are at odds with an instrumental relationship to the organization (Albdour and Altarawneh, 2014). In other words, although work-engaged individuals may derive self-fulfillment from their work role, this does not necessarily mean that their motivation is grounded in pursuing individual success and achievements. A collective orientation is instead more likely to play a significant role in driving work engagement. This idea is supported by robust evidence indicating that transformational leadership, notably characterized by an emphasis on shared interests and collective goals, is a catalyst for work engagement (e.g., Lai et al., 2020). Overall, our results are consistent with Roney and Soicher (2021) recent suggestion that higher levels of individual self-concept may trigger negative forms of work investment (i.e., workaholism). An intriguing and unexpected finding was however that workaholism was related to stronger collective self-concept. In retrospect, such relationship might be explained by the fact that by working (too) hard, individuals expect to serve the cause of the organization (i.e., the hallmark of the collective), as presumably strong achievements at work may contribute to its performance, though empirical research invalidates such effect of workaholism (Balducci et al., 2020). This neglected aspect of workaholism could be further examined in future research.

Turning to the outcomes, our results lend support for a clear differentiation between bright (i.e., work engagement and, to a lesser extent, affective commitment) and dark (i.e., workaholism) forms of work investment (Di Stefano and Gaudiino, 2019). Two aspects warrant specific attention. First, controlling for workaholism, work engagement, and affective commitment baseline levels, we found that only workaholism predicted increases in work hours and role overload. Robust evidence for the positive relationship between workaholism, work hours, and role overload has already accumulated [see Clark et al. (2016) for a meta-analysis]. Yet, because this evidence stems from cross-sectional studies, the direction of the relationships remained uncertain (Clark et al., 2016). Our work corroborates the idea that workaholics either create extra work to fulfill their work drive, hence explaining their propensity to work long hours, or overestimate their workload due to a self-serving attribution bias (Clark et al., 2016; Snir and Harpaz, 2012). Future research that measures objective workload is needed to assess the accuracy of either one or both explanations. Relatedly, our findings suggest that work engagement and affective commitment do not manifest through long hours and additional work. However, we cannot rule out the possibility that, unlike workaholics, work-engaged and affectively committed employees underestimate their investment in work. Moreover, these dynamics may depend on workers’ characteristics and context: for example, men and workers in managerial positions may be more inclined to demonstrate their engagement and their commitment through long hours and additional work because these behaviors are particularly valued among these groups (Messenger, 2018). Future research may explore this avenue.

Regarding emotional exhaustion and depression, work engagement appears to be more protective than affective commitment. These conclusions align with earlier cross-sectional analyses by Hallberg and Schaufeli (2006) indicating that work engagement was more negatively associated with health complaints, including emotional exhaustion and depression, than overall organizational commitment. Our research extends these findings by not only establishing the directionality of this relationship with a time-lagged design, but also by highlighting the superior protective role of engagement over the affective dimension of commitment. The positive impact of work engagement on mental health may be attributed to the positive experiences arising from this psychological state (i.e., positive emotions) or to its strong association with individual dispositions that are known to offer protection against mental health issues, whereas affective commitment has been shown to primarily stem from the work context (Mazzetti et al., 2021b; Meyer et al., 2002).

Finally, the fact that workaholism was found to predict heightened levels of depression but not emotional exhaustion is intriguing as meta-analytic findings showed a positive correlation between the latter and workaholism (Clark et al., 2016). Methodological factors might explain this absence of a significant relationship. Although the question is still unresolved, it’s been indeed suggested that work-related depression precedes emotional exhaustion (Koutsimani et al., 2019), a scenario that, if applicable to our study, could elucidate our findings. However, amidst the ongoing debate on the overlap between burnout and depression (Koutsimani et al., 2019), our results at least underscore the distinction between these two forms of distress.

### Limitations and future directions

Despite its strengths, our research has limitations worth noting. First, all the data were from a single source, e.g., employees, which may have inflated associations between the variables due to common method bias (Podsakoff et al., 2012). We, however, employed temporal separation and controlled for autoregressive effects to minimize inflated covariations. Nonetheless, the fact that respondents provided self-reported information may have biased the findings due to incorrect recall, desirability distortion, or subjective interpretation. For instance, research indicates that estimates of work hours are especially prone to inaccurate reports (Ganster et al., 2016). Workaholics may tend to overestimate their hours worked while work-engaged and affectively committed employees may tend to underestimate them. Subjective role overload ratings may be subject to similar distortions. Our findings should therefore be replicated using objective measures of time spent working.

Second, we cannot rule out the possibility that socio-economic and/or cultural factors may have influenced our results, given that our sample predominantly consisted of highly educated employees primarily employed in France, with a substantial proportion (62%) holding managerial positions. For example, the average working week within our sample was 49 h, contrasting with the national average of 35 to 40 h per week (DARES, 2024). In 2023, the French national average was 38.9 h worked per week, while the average was slightly higher for executives (42.1 h) (INSEE, 2023). Similarly, European surveys report that the average number of hours worked per week in France and across Europe was 36 in 2023 (Eurostat, 2024). Compared to these numbers, the average number of hours worked per week reported in our sample is pretty high. This may explain the absence of a significant relationship between work engagement, affective commitment, and work hours, as employees may have been unable to further extend their already long work hours. Future studies may thus explore if our results remain valid among less educated workers in other countries. Work engagement and affective commitment may indeed exhibit different patterns of associations among countries where a strong Protestant work ethic prevails (e.g., the US), such that work and morality are closely intertwined (D’Iribarne, 1989). In such countries, work hours may exhibit a stronger association with engagement and commitment. Third, it is worth mentioning that we collected our data before the COVID-19 pandemic and the widespread adoption of hybrid and remote work. Because evidence accumulates on the singularity of work dynamics and investment in remote settings (e.g., Bareket-Bojmel et al., 2023), future studies should examine the patterns of association between types of heavy work investment and work and personal outcomes in remote work contexts.

Third, we encourage researchers to further explore the distinctions between the various forms of HWI. For instance, future studies could build on our work by examining the boundaries between workaholism, work engagement, affective commitment, and related constructs such as obsessive and harmonious passion (Vallerand et al., 2003). While existing empirical evidence supports the distinctiveness of these concepts (e.g., Birkeland and Buch, 2014), their interrelations remain unclear. Some researchers have proposed that passion may serve as an antecedent to HWI rather than a parallel construct (Tóth-Király et al., 2020), an avenue that warrants further investigation.

### Practical implications

Our findings are of importance for practitioners in two important ways. First, we suggest that working patterns characterized by hard work, even if not easily distinguishable for managers, should nonetheless be recognized. Because workaholism is associated with deleterious consequences on workers’ health and considering the mounting evidence showing its lack of association with enhanced performance (e.g., Balducci et al., 2020), managers should learn to recognize the syndrome and monitor workaholics’ work hours and workload. In contrast, since work engagement appears to be the most protective form of work investment, managers should be trained to implement practices that help promote this form of heavy work investment, especially because leaders have been found to play a critical role in sustaining work engagement (Bakker and Albrecht, 2018). Second, meetings to discuss employees’ motivation could help managers identify those self-oriented employees who are at a higher risk of becoming workaholics. Managers could then aim to redirect the behaviors of these employees toward constructive work behaviors, for example by emphasizing work achievements that require quality rather than quantity work.

## Conclusion

This study reveals that workaholism, work engagement, and affective commitment, three psychological states that fall under the umbrella of heavy work investment, are distinct. The results indicate that employees’ levels of self-concepts differentially relate to the three forms of work investment, with the individual self-concept being uniquely associated with workaholism. Moreover, our study provides further evidence for the clear distinction between workaholism as a dark type of heavy work investment and work engagement as a bright counterpart. While the former is associated with poorer mental health, the latter is linked to enhanced mental well-being. Affective commitment is not associated with changes in either depression or emotional exhaustion. These findings suggest efforts should be directed at promoting work engagement and minimizing the prevalence of workaholism.

## Data Availability

The raw data supporting the conclusions of this article will be made available by the authors, without undue reservation.
